# Long-Term Volumetric Stability of Maxillary Sinus Floor Augmentation Using a Xenograft Bone Substitute and Its Combination with Autologous Bone: A 6+ Year Retrospective Follow-Up Study Using Cone Beam Computed Tomography

**DOI:** 10.3390/dj12050121

**Published:** 2024-04-24

**Authors:** Liene Zamure-Damberga, Oskars Radzins, Girts Salms, Maksims Zolovs, Zanda Bokvalde, Laura Neimane

**Affiliations:** 1 Department of Conservative Dentistry and Oral Health, Riga Stradins University, LV-1007 Riga, Latvia; oskars.radzins@rsu.lv (O.R.); zanda.bokvalde@rsu.lv (Z.B.); laura.neimane@rsu.lv (L.N.); 2 RSU Institute of Stomatology, LV-1007 Riga, Latvia; girts.salms@rsu.lv; 3 Baltic Biomaterials Centre of Excellence, Headquarters at Riga Technical University, LV-1073 Riga, Latvia; 4 Department of Oral and Maxillofacial Surgery, Riga Stradins University, LV-1007 Riga, Latvia; 5 Statistics Unit, Riga Stradins University, LV-1048 Riga, Latvia; maksims.zolovs@rsu.lv; 6 Institute of Life Sciences and Technology, Daugavpils University, LV-5401 Daugavpils, Latvia

**Keywords:** maxillary sinus floor augmentation, xenograft, deproteinised bovine bone, long-term study, CBCT

## Abstract

Deproteinised bovine bone (DBB) is widely used as bone substitute in maxillary sinus floor augmentation (MSFA) surgery. No previous studies have shown the long-term volumetric changes in the augmented bone when using DBB. The selected patients had MFSA performed using a lateral window technique and a xenograft, alone or in combination with the patient’s autologous bone from the mandible. Cone beam computed tomography (CBCT) images were used to compare the volumetric changes in the augmented bone for patients over a period of 6 or more years. No significant bone reduction was seen in the augmented bone region when comparing MSFA after 7 months and 6 or more years after dental implantation.

## 1. Introduction

Replacement of missing or lost teeth with dental implants is a choice many patients make today. Still, teeth loss in the alveolar processes leads to bone reduction and insufficient bone volume, which can be a factor that negatively affects the outcome of dental implantation. If the vertical bone dimension in the maxilla is reduced, surgeons can opt for a short dental implant, with recent studies confirming this to be a viable solution in the long term [1], or maxillary sinus floor augmentation (MSFA) surgery with a bone substitute [2]. Usually, the bone can be substituted with the patient’s autologous bone, a xenograft bone substitute from deproteinised bovine bone (DBB), synthetic materials, or a combination of these. Yet, a systematic review and meta-analysis by Starch-Jensen et al. and a study by Sakkas et al. both suggest that significant volumetric stability can be obtained by using a xenograft or mixing it with autologous bone when compared with the previous “gold standard”—autologous bone [3,4]. Xenografts are characterised by their significantly good clinical outcomes [5]. For more successful outcomes, surgeons use barrier membranes so soft tissue does not infiltrate the area where bone regeneration should happen. As described in another systematic review, barrier membranes also increase the percentage of newly formed bone and prevent the displacement of grafting material, so using a barrier membrane can directly impact the stability, volume, and structure of the grafted bone. [6]. There are resorbable and non-resorbable membranes that can be used, and each has to be chosen in consideration of the functional requirements of the specific clinical application [7,8].

Cone beam computed tomography (CBCT) is a commonly used radiological modality in the maxillofacial region for bone augmentation, implantation, and other surgical procedures to evaluate the anatomy of bone in all dimensions [9]. CBCT, as with any radiological examination, has limitations that can affect the evaluation of images, like density assessment and artefacts caused by metallic objects, which must be considered when choosing the examination parameters for the patient and inspecting the images acquired [9,10,11]. 

The scientific value of volumetric stability of MSFA is to show the bone graft behaviour, as it is possible to describe not only the changes in bone height but also its quality and density, which cannot be observed in less advanced imaging modalities. Understanding the behaviour of a bone graft in the long term can help clinicians choose the best augmentation material for the intended application.

The purpose of this retrospective study was to radiologically evaluate the volumetric changes in the augmented bone in the maxilla with a xenograft, or its combination with autologous bone, in the long term using CBCT images. No such studies have been conducted previously, as far as is known by the authors.

## 2. Materials and Methods

This retrospective radiological study was performed at the Riga Stradins University (RSU) Institute of Stomatology, and all the data acquisition took place between 2015 and 2018. The study was performed following the Declaration of Helsinki (2013 revision) [12], and it was conducted with the approval of the Ethics Committee of RSU (Nr.12/10.09.2015).

### 2.1. Patient Selection Criteria

Manual patient selection was performed by reviewing the surgery journals between 28 November 2007 and 1 December 2012. All consecutive patients who had dental implantation with bone augmentation were included. Then, the radiological examination database was checked to see if the patients had a CBCT examination performed after the bone augmentation, but before dental implantation. The next inclusion criteria were to check corresponding databases to see if the patient was not deceased and that they are residents of Latvia. The patient’s internal medical record had to be completed with all necessary information about all the surgical procedures (the material used, the procedure approach, dates and details about the implantation). One hundred forty-six patients were invited for a control visit and a CBCT examination. Out of the 146 patients who were invited for a control visit and a CBCT examination, there were 59 responses from whom informed consent was obtained to participate in the study. Patients with bone augmentation in the lower jaw or horizontal ridge augmentation and those with synthetic bone substitutes or allografts and their combinations were excluded from this study. The remaining 14 patients (16 sinuses) with MSFA with xenograft or its combination with autologous bone and who had a CBCT examination with minimal distortion within the augmented bone area were included in the study. No study patients had sinusitis, ostium blockage, hemosinus, or implant displacement; the implants were stable.

All the CBCT scans used for this study were performed with i-CAT (Next Generation, Imaging Science, Hatfield, PA, USA). Images were taken with a voxel size of 0.3 mm using 120 kV, 5 mA, and an exposure time of 4 s.

### 2.2. Maxillary Sinus Floor Augmentation (MSFA)

Sinus floor augmentation surgeries were indicated for patients for whom the residual bone height before the surgery was 5 mm or less [13]. Unfortunately, there was no information within patient records on whether they were smokers. According to the protocol in the clinic, it is a contraindication for bone augmentation and implant surgery if the patient is a heavy smoker (smoking more than 20 cigarettes per day). The surgeries for the study patients were performed using xenograft granules (BioOss^®^, Geistlich Pharm AG, Wolhausen, Switzerland) alone or in combination with patients’ autologous bone from the mandible. The surgery was performed using the lateral window technique. None of the study patients had reported health conditions that could have affected bone augmentation or dental implant survival (such as osteoporosis, diabetes, hypothyroidism, or cardiovascular disease). The surgical procedures were performed by several surgeons using the same technique in the same clinic. For some patients, membranes were used, as can be seen in Table 1.

### 2.3. Evaluation of Volumetric Change

The scans were examined by two examiners (LZD and OR) in agreement with all the assessments, and measurements and consensus results were used for statistical analysis.

First, a voxel-based superimposition using the cranial base as the reference region was performed for each patient. The maxillary region containing the augmented area was excluded from the reference for improved superimposition results. Then, the same volume of interest (VOI) was manually selected in images from both timepoints and subsequently extracted from the superimposed images for further processing.

**Figure 1:**
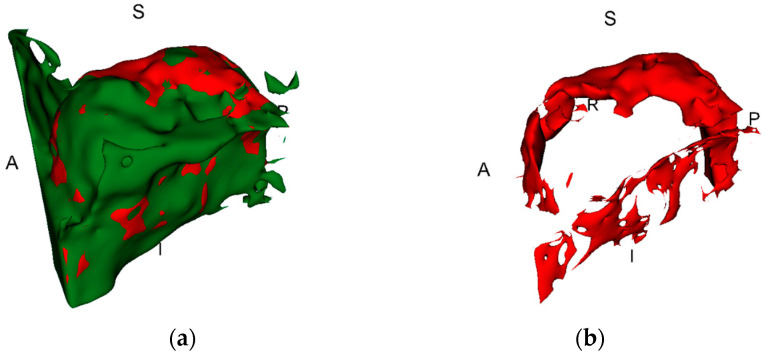
The green area shows all the tissues of augmented bone region together with artefacts and hard tissue components from the maxillary sinus, while the red area shows the true change in bone volume; (**a**) T1 together with T2; (**b**) the change in bone before manual correction of artefacts.

This was followed by a two-step segmentation process within the VOI to create a representation of the augmented region. Firstly, by thresholding, the lower and upper-value limits (usually within a range of 100–200 grey values for the lower bound and 800–1000 for the upper bound) were set individually for each case to obtain optimal signal-to-noise ratio. The limits were chosen in agreement between the two experts and secondly, by manually correcting the augmented region to remove any artefacts created by the implants and adjusting the surface of the augmented region if necessary. This is visualised in Figure 1. It was assumed that the augmentation material is consistent throughout the reconstructed region. Therefore, to obtain volumetric measurements, any holes within the boundary of the augmented area were filled. The same segmentation process was used for both timepoints.

Once segmentations were created, a Boolean subtraction was performed to create the region indicative of volumetric change. The bone volume at T2 was subtracted from T1, considering the augmented bone region decreases in volume over time.

This region was manually edited to remove any components that did not contribute to the region of interest (ROI). In this study, the ROI was the change in the augmented bone fragment, not the whole maxillary segment. Once finalized, the volumetric value descriptive of the change in volume over time was recorded and named as the true change in bone volume. Figure 1 illustrates the bone changes in the ROI and the true changes in bone volume.

Before this study, both experts were calibrated. The same two experts performed all segmentations and measurements in complete consensus.

We identified the volume measurements in time as T1—the volume of augmented bone after the augmentation surgery and before implantation and the baseline examination—whereas T2 is the volume of augmented bone in the six-year or longer follow-up.

### 2.4. Statistical Analysis

The assumption of data distribution was assessed using the Shapiro–Wilk test and inspection of the normal Q-Q plots. Bone volume at T1 and T2 was compared using the Wilcoxon rank test. The true volume change without artefacts was compared with bone volume at T1 and T2 using the Mann–Whitney U test. The difference between the bone volume at T1 and T2 was calculated and compared with the true change in volume without artefacts. Also, the Mann–Whitney U test was used to compare bone volume at T1 and T2 and the true change in volume without artefacts between materials (xenograft (BioOss^®^) and xenograft (BioOss^®^) combination with autologous bone). The Kruskal–Wallis H test was used to compare bone volume at T1 and T2 and the true change in volume without artefacts between membranes (titanium mesh membrane, BioGide^®^ collagen membrane, and no membrane used). The statistical analyses were performed using the Jamovi program [14]. Differences were considered statistically significant at *p* < 0.05.

## 3. Results

The average time between T1 and T2 was 6.6 years (344 weeks). The average age of the patients was median = 50 and IQR 42–56 years at T1 and median = 56.5 and IQR 50–62 years at T2. The study group consisted of thirteen women and seven men. The median time between the MSFA surgery and T1 CBCT was 217 (Q1–Q3 188–286) days or 31 weeks or 7.13 months. Table 1 includes the descriptive statistics of the patients and information about the materials and membranes used.

The difference between the bone volume at T1 and T2 was the bone volume that included the components that did not contribute to the ROI for this study. Table 1 shows the median of true bone changes to be 2% or 230.5 mm^3^.

**Figure 2:**
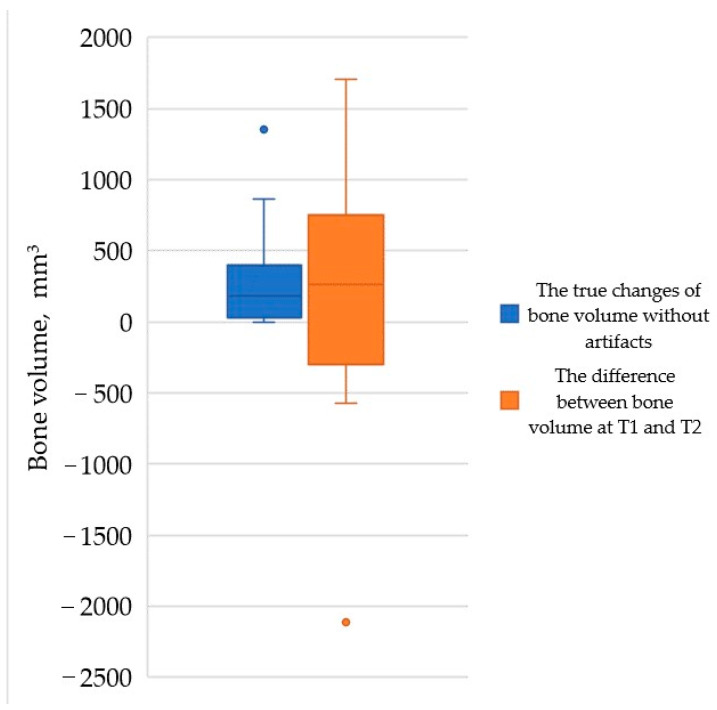
The median with interquartile range (Q1–Q3) shows the bone volume difference between true changes in volume without artefacts and the difference between the bone volume at T1 and T2.

No statistically significant differences were found in bone volume at T1 and T2 (*p* > 0.05). Additionally, there were no statistically significant differences between the bone volume changes at T1 and T2 compared to the true changes in volume without artefacts (*p* > 0.05). Furthermore, no statistically significant differences were observed in bone volume at T1 and T2 or in the true changes in volume without artefacts between materials (xenograft or xenograft combination with autologous bone) and membranes (no membrane, titanium mesh membrane, and collagen membrane) (*p* > 0.05). Figure 2 illustrates the difference between the measurements.

## 4. Discussion

**Figure 3:**
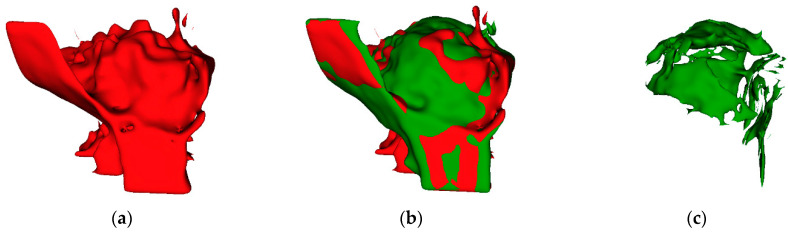
Visualisation of alveolar bone and the augmented region (ROI). (**a**) T2 with large artefacts below alveolar process; (**b**) T1 (green) overlapped with T2 (red); (**c**) the true change in augmented bone from T1 to T2.

The difference between bone volume at T1 and T2 was positive and negative, which are indicative of bone volume increase or decrease, respectively. This measurement only shows the volume close to the ROI, which in this study, was the actual augmented material area, and it could contain artefact shadows that increased the measured volume next to the augmented bone, nor did it indicate the true volume change. It was used only to visualize the difference and the number of artefacts. Figure 3 shows the comparison of the alveolar process in different timepoints from the same point of view.

In this study, the T1 examination was ordered by the operating surgeon to see the augmented bone after the healing process, which was followed by implantation soon after. Because the average time from MSFA until T1 CBCT was approximately 7 months, relying on other studies, it is possible to speculate about the bone resorption that had happened up to that point. A similar study by Mazzocco et al. also studied bone volume changes in MSFA with xenograft granules in the first 8 months, showing bone reduction of approximately 10% [15]. A systematic review by Shanbhag et al. concluded that synthetic bone substitutes or xenografts, alone or in combination with autogenous bone, showed an average volume decrease by approximately 18–23%, all in shorter-term studies [16]. It is possible to speculate with this data that similar bone reduction must have happened to the patients in the present study group in the earlier postoperative period.

This research only studies bone augmentations using a xenograft or its combination with autologous bone from the mandible. A different group using an alternative augmented material for comparison was not used in this study. In 2011, Jensen et al. in their systematic review, noticed a lack of studies about volumetric changes when a xenograft or its combination with autologous bone was used [5]. However, over the years, research on this topic has improved. A systematic review showed that the volumetric stability of the grafting material following maxillary sinus floor augmentation was significantly improved using a mixture of an autogenous bone graft and a xenograft compared to an autogenous bone graft alone. They state that the degree of volume change might be influenced by the patient’s individual characteristics and the chemical and physical properties of the grafting material itself [3]. Other systematic reviews state that most volume reductions happened during the first years after the MSFA, regardless of the material type or the combinations used [17,18,19]. Similar to the barrier membranes—the clinical outcome might depend more on the surgeons’ skills and expertise rather than on the membrane type used [8]. However, even if the barrier membranes do not directly impact the survival of the implant, they can improve the stability of the grafted bone [6]. So, it is possible that the comparison of different materials is secondary, and more attention should be paid to the patients’ individual parameters and/or to the surgeons’ skills than to the materials used. 

Another aspect of the study that was not evaluated was the amount of material used. In a systematic review by Pesce et al., it was concluded that the amount of grafting material used can affect its behaviour, meaning that it is important to avoid excessive hyper-augmentation to obtain the best stability and quality of the augmented bone [20].

There are just a few studies that have researched long-term graft change after MSFA. Hatano et al., in their study, described minimal bone reduction in augmentations with a xenograft in a subsequent 2- to 3-year period back in 2004; however, the measurements were performed using panoramic imaging [21]. There is plenty of high-quality research on the marginal bone level changes in long-term studies [22,23,24,25,26], which is important because it shows the change in bone that is in close contact with the outer environment, but it does not show the changes that the graft itself undergoes. Other studies showed bone volume changes in the long term, but these studies were mostly about autologous bone grafts [27,28,29].

The results obtained showed that using a xenograft on its own or combined with autologous bone led to a very low bone volume change in the long term, which coincides with clinical biopsy results that show slow resorption rates after 6 or more years [30]. Furthermore, it has been shown histologically that bone augmentation using a xenograft leads to vital and mature bone formation in the long term, so it becomes capable of withstanding loading forces. The lack of chronic inflammatory cell infiltration or other adverse effects and excellent implant survival rates have also been shown in other histological studies [31,32]. Clinically, this could mean that if a dental implant has survived the early postoperative period, there is very little chance that there would be any significant changes in the graft itself over a prolonged period of time; therefore, the implant success will solely depend on the external factors, such as the prosthodontic plan and patient’s oral health.

There are also different examination methods that can be used to evaluate the bone structure and mineralisation that show even more detailed information from histological samples, like environmental scanning electron microscopy (ESEM) and energy dispersive X-ray spectroscopy (EDX). Results in shorter-term studies indicate that the bone structure 9 months after augmentation might not yet be ready for the application of loading forces due to bone mineralisation not being complete throughout the augmented area [33]. This could be a very interesting prospect for the future for examining the bone in the long term in a similar way. 

**Figure 4:**
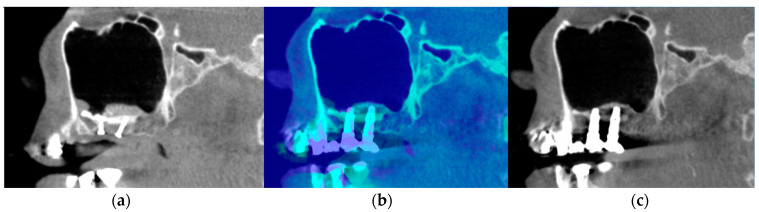
Sagittal view of augmented sinus in different timepoints. (**a**) Sagittal view of augmented sinus in the T1 timepoint, with fixation screws under the augmented region visible; (**b**) both timepoints overlapped with colour enhancement; (**c**) sagittal view of the augmented sinus in T2 with a large artefact impact in area between both implants in the augmented bone region.

The first limitation of the study is the group size, which is smaller than the obtained CBCTs from the initial study. This happened because many examinations showed a large artefact over the ROI, and it was impossible to visualise the bone, so they had to be excluded. Figure 4 illustrates the impact of artefacts in T2 and how the bone looked previously in T1.

This problem could possibly be reduced by increasing the kV during the exposure [34], but this would also lead to an increase in the patients’ received radiation dosage. Using a scanning protocol with higher kV to have reduced artefact impact on the CBCT scan, if not for true diagnostic purposes, would not be ethical [35]. Another limitation is that this study is solely retrospective, and the results included do not show the implant survival or success rates, as this study was conducted only to compare the bone volume changes in the CBCT images.

Many studies on bone volume change in MSFA over the long term have been conducted in the previous decades, and the majority of them are about autologous bone changes. Yet we have not found a radiological study similar to the present research that would visualise and analyse three-dimensional changes over such a long term in which xenograft materials would have been used.

## 5. Conclusions

Within the limits of this study, xenograft granules used in MSFA are a stable and predictable material. Only very small volume changes can be seen in regions augmented with a xenograft alone or combined with autogenous bone in a 6-year or longer follow-up. Similar comparison in the long term should be researched to compare larger sample sizes and include the data on unsuccessful implantations and implant failures.

## Figures and Tables

**Table 1:** Characteristics of study group population, measurements of bone volume, and calculation results.

Patient ID	Sex	Age at T1 (years)	Days between MFBA and T1	Material Used for MFBA	Membrane	Bone Volume at T1 (mm^3^)	Bone Volume at T2 (mm^3^)	T1-T2 (Including Artefacts) *	True Bone Volume Change (mm^3^)	True Bone Volume Change (%)
1	F	57	236	XG	Ti	7908	6204	1704	1351	17%
2	M	54	159	XG + AB	-	4900	3977	923	477	10%
3	F	30	273	XG	Ti	6333	5529	804	862	14%
4	F	41	205	XG + AB	Ti	3192	3767	−575	0	0%
5	F	41	205	XG + AB	Ti	3643	3437	206	198	5%
6	F	50	349	XG + AB	-	6116	5536	580	263	4%
7	F	51	680	XG	-	4655	5005	−350	0	0%
8	M	58	313	XG	Ti	4495	4653	−158	39	1%
9	F	38	353	XG	Ti	3526	2927	599	317	9%
10	F	53	189	XG	-	5002	5135	−133	165	3%
11	F	56	277	XG + AB	-	5817	4276	1541	423	7%
12	F	39	185	XG	BG	3947	3626	321	53	1%
13	M	49	217	XG	BG	23,688	25,802	−2114	119	1%
14	M	49	217	XG	BG	23,688	25,802	−2114	304	1%
15	M	47	138	XG	Ti	5169	4743	426	17	0%
16	F	56	167	XG	Ti	4763	4831	−68	23	0%
		Median:	217		Median:	4951	4787	263.5	181.5	2%
		Q1–Q3:	188–286		Q1–Q3:	4358–6170	3924–5531	−302–752.75	35–344	

* Negative value in the difference between T1 and T2 would technically indicate an increase in bone volume but actually indicates a large volume of artifact overlapping the ROI. XG—xenograft (BioOss^®^). AB—autologous bone; Ti—titanium mesh membrane; BG—collagen membrane (BioGide^®^ Geistlich Pharm AG, Wolhausen, Switzerland); M—male; F—female.

## Data Availability

The datasets used and analysed during the current study are presented in the results section. Some data are unavailable due to privacy and ethical restrictions.

## References

[1] Iezzi G., Perrotti V., Felice P., Barausse C., Piattelli A., Del Fabbro M. (2020). Are <7-mm long implants in native bone as effective as longer implants in augmented bone for the rehabilitation of posterior atrophic jaws? A systematic review and meta-analysis. Clin. Implant. Dent. Relat. Res..

[2] Boyne P.J., James R.A. (1980). Grafting of the maxillary sinus floor with autogenous marrow and bone. J. Oral Surg..

[3] Starch-Jensen T., Deluiz D., Vitenson J., Bruun N.H., Tinoco E.M.B. (2021). Maxillary Sinus Floor Augmentation with Autogenous Bone Graft Compared with a Composite Grafting Material or Bone Substitute Alone: A Systematic Review and Meta-Analysis Assessing Volumetric Stability of the Grafting Material. J. Oral Maxillofac. Res..

[4] Sakkas A., Wilde F., Heufelder M., Winter K., Schramm A. (2017). Autogenous bone grafts in oral implantology-is it still a “gold standard”? A consecutive review of 279 patients with 456 clinical procedures. Int. J. Implant. Dent..

[5] Jensen T., Schou S., Stavropoulos A., Terheyden H., Holmstrup P. (2012). Maxillary sinus floor augmentation with Bio-Oss or Bio-Oss mixed with autogenous bone as graft: A systematic review. Clin. Oral Implant. Res..

[6] Starch-Jensen T., Deluiz D., Duch K., Tinoco E.M.B. (2019). Maxillary Sinus Floor Augmentation With or Without Barrier Membrane Coverage of the Lateral Window: A Systematic Review and Meta-Analysis. J. Oral Maxillofac. Res..

[7] Rakhmatia Y.D., Ayukawa Y., Furuhashi A., Koyano K. (2013). Current barrier membranes: Titanium mesh and other membranes for guided bone regeneration in dental applications. J. Prosthodont. Res..

[8] Zhang M., Zhou Z., Yun J., Liu R., Li J., Chen Y., Cai H., Jiang H.B., Lee E.S., Han J. (2022). Effect of Different Membranes on Vertical Bone Regeneration: A Systematic Review and Network Meta-Analysis. Biomed Res. Int..

[9] Codari M., de Faria Vasconcelos K., Ferreira Pinheiro Nicolielo L., Haiter Neto F., Jacobs R. (2017). Quantitative evaluation of metal artifacts using different CBCT devices, high-density materials and field of views. Clin. Oral Implant. Res..

[10] Pauwels R., Jacobs R., Singer S.R., Mupparapu M. (2015). CBCT-based bone quality assessment: Are Hounsfield units applicable?. Dentomaxillofac. Radiol..

[11] Fontenele R.C., Nascimento E.H., Vasconcelos T.V., Noujeim M., Freitas D.Q. (2018). Magnitude of cone beam CT image artifacts related to zirconium and titanium implants: Impact on image quality. Dentomaxillofac. Radiol..

[12] Association W.M. (2013). World Medical Association Declaration of Helsinki: Ethical principles for medical research involving human subjects. Jama.

[13] Misch C.E. (1987). Maxillary sinus augmentation for endosteal implants: Organized alternative treatment plans. Int. J. Oral Implantol..

[14] Jamovi (2022). The Jamovi Project (Version 2.3) [Computer Software]. https://www.jamovi.org.

[15] Mazzocco F., Lops D., Gobbato L., Lolato A., Romeo E., del Fabbro M. (2014). Three-dimensional volume change of grafted bone in the maxillary sinus. Int. J. Oral Maxillofac. Implant..

[16] Shanbhag S., Shanbhag V., Stavropoulos A. (2014). Volume changes of maxillary sinus augmentations over time: A systematic review. Int. J. Oral Maxillofac. Implant..

[17] Starch-Jensen T., Aludden H., Hallman M., Dahlin C., Christensen A.E., Mordenfeld A. (2018). A systematic review and meta-analysis of long-term studies (five or more years) assessing maxillary sinus floor augmentation. Int. J. Oral Maxillofac. Surg..

[18] Raghoebar G.M., Onclin P., Boven G.C., Vissink A., Meijer H.J.A. (2019). Long-term effectiveness of maxillary sinus floor augmentation: A systematic review and meta-analysis. J. Clin. Periodontol..

[19] Nkenke E., Stelzle F. (2009). Clinical outcomes of sinus floor augmentation for implant placement using autogenous bone or bone substitutes: A systematic review. Clin. Oral Implant. Res..

[20] Pesce P., Menini M., Canullo L., Khijmatgar S., Modenese L., Gallifante G., Del Fabbro M. (2021). Radiographic and Histomorphometric Evaluation of Biomaterials Used for Lateral Sinus Augmentation: A Systematic Review on the Effect of Residual Bone Height and Vertical Graft Size on New Bone Formation and Graft Shrinkage. J. Clin. Med..

[21] Hatano N., Shimizu Y., Ooya K. (2004). A clinical long-term radiographic evaluation of graft height changes after maxillary sinus floor augmentation with a 2:1 autogenous bone/xenograft mixture and simultaneous placement of dental implants. Clin. Oral Implant. Res..

[22] Park W.B., Han J.Y., Kang K.L. (2021). Long-Term Comparison of Survival and Marginal Bone of Implants with and without Sinus Augmentation in Maxillary Molars within the Same Patients: A 5.8- to 22-Year Retrospective Study. J. Clin. Med..

[23] Sbordone C., Toti P., Ramaglia L., Guidetti F., Sbordone L., Martuscelli R. (2014). A 5-year clinical and computerized tomographic implant follow-up in sinus-lifted maxillae and native bone. Clin. Oral Implant. Res..

[24] Sbordone C., Toti P., Martuscelli R., Guidetti F., Sbordone L., Ramaglia L. (2015). A 5-Year Implant Follow-Up in Maxillary and Mandibular Horizontal Osseous Onlay Grafts and Native Bone. J. Oral Implantol..

[25] Hallman M., Zetterqvist L. (2004). A 5-year prospective follow-up study of implant-supported fixed prostheses in patients subjected to maxillary sinus floor augmentation with an 80:20 mixture of bovine hydroxyapatite and autogenous bone. Clin. Implant. Dent. Relat. Res..

[26] Nyström E., Ahlqvist J., Gunne J., Kahnberg K.E. (2004). 10-year follow-up of onlay bone grafts and implants in severely resorbed maxillae. Int. J. Oral Maxillofac. Surg..

[27] Nyström E., Ahlqvist J., Legrell P.E., Kahnberg K.E. (2002). Bone graft remodelling and implant success rate in the treatment of the severely resorbed maxilla: A 5-year longitudinal study. Int. J. Oral Maxillofac. Surg..

[28] Sbordone C., Toti P., Guidetti F., Califano L., Santoro A., Sbordone L. (2012). Volume changes of iliac crest autogenous bone grafts after vertical and horizontal alveolar ridge augmentation of atrophic maxillas and mandibles: A 6-year computerized tomographic follow-up. J. Oral Maxillofac. Surg..

[29] Cansiz E., Haq J., Manisali M., Cakarer S., Gultekin B.A. (2020). Long-term evaluation of three-dimensional volumetric changes of augmented severely atrophic maxilla by anterior iliac crest bone grafting. J. Stomatol. Oral Maxillofac. Surg..

[30] Schlegel A.K., Donath K. (1998). BIO-OSS—A resorbable bone substitute?. J. Long Term Eff. Med. Implant..

[31] Iezzi G., Degidi M., Scarano A., Petrone G., Piattelli A. (2007). Anorganic bone matrix retrieved 14 years after a sinus augmentation procedure: A histologic and histomorphometric evaluation. J. Periodontol..

[32] Ferreira C.E., Novaes A.B., Haraszthy V.I., Bittencourt M., Martinelli C.B., Luczyszyn S.M. (2009). A clinical study of 406 sinus augmentations with 100% anorganic bovine bone. J. Periodontol..

[33] Imai H., Prati C., Zamparini F., Iezzi G., Botticelli D., Gandolfi M.G., Baba S. (2023). ESEM-EDX Mineralization and Morphological Analysis of Human Retrieved Maxillary Sinus Bone Graft Biopsies before Loading. J. Funct. Biomater..

[34] Demirturk Kocasarac H., Koenig L.J., Ustaoglu G., Oliveira M.L., Freitas D.Q. (2022). CBCT image artefacts generated by implants located inside the field of view or in the exomass. Dentomaxillofac. Radiol..

[35] Ludlow J.B., Timothy R., Walker C., Hunter R., Benavides E., Samuelson D.B., Scheske M.J. (2015). Effective dose of dental CBCT-a meta analysis of published data and additional data for nine CBCT units. Dentomaxillofac. Radiol..

